# Species delimitation and coexistence in an ancient, depauperate vertebrate clade

**DOI:** 10.1186/s12862-022-02043-4

**Published:** 2022-07-12

**Authors:** Chase Doran Brownstein, Immanuel Chas Bissell

**Affiliations:** 1 Stamford Museum and Nature Center, Stamford, CT USA; 2 Department of Ecology and Evolutionary Biology, Yale University, New Haven, CT USA; 3 Department of Earth and Planetary Sciences, Yale University, New Haven, CT USA

**Keywords:** Coelacanths, Diversity, Speciation, Paleontology, Triassic

## Abstract

**Background:**

A major challenge to understanding how biodiversity has changed over time comes from depauperons, which are long-lived lineages with presently low species diversity. The most famous of these are the coelacanths. This clade of lobe-finned fishes occupies a pivotal position on the vertebrate tree between other fishes and tetrapods. Yet only two extant species and fewer than 100 extinct forms are known from the coelacanth fossil record, which spans over 400 million years of time. Although there is evidence for the existence of additional genetically isolated extant populations, a poor understanding of morphological disparity in this clade has made quantifying coelacanth species richness difficult.

**Results:**

Here, we quantify variation in a sample of skulls and skeletons of the Triassic eastern North American coelacanth †*Diplurus* that represents the largest assemblage of coelacanth individuals known. Based on the results of these quantitative comparisons, we identify a diminutive new species and show that multiple lacustrine ecosystems in the Triassic rift lakes of the Atlantic coastline harbored at least three species of coelacanths spanning two orders of magnitude in size.

**Conclusions:**

Conceptions about the distribution of species diversity on the tree of life may be fundamentally misguided when extant diversity is used to gauge signals of extinct diversity.

Our results demonstrate how specimen-based assessments can be used to illuminate hidden biodiversity and show the utility of the fossil record for answering questions about the hidden richness of currently species-poor lineages.

**Supplementary Information:**

The online version contains supplementary material available at 10.1186/s12862-022-02043-4.

## Introduction

Major changes to biodiversity over the history of life on Earth have shaped extant species richness [1, 68, 78]. Although one end of the spectrum of biodiversity-radiations-are a historically well-studied evolutionary phenomenon [1, 31, 33, 75], the origins of species-poor lineages that have persisted for millions or tens of millions of years are gaining attention (e.g., [22, 23, 57]. Phylogenomic studies now recognize depauperons across of different portions of the Tree of Life (e.g., [2, 10, 40, 42, 66, 89]), demonstrating that depauperacy is a consistent evolutionary pattern.

Nonetheless, the fossil records of many currently species-poor clades show how a view of the evolutionary history of these clades based solely on extant forms is often biased. Among vertebrates, clades now represented by one or a handful of species are represented by numerous species with varying morphologies in the fossil record [35–38, 57, 77]. This discrepancy between extant and extinct species richness and disparity in depauperons means that rigorous species delimitation using quantitative methods is particularly important for properly detecting their diversity and understanding their evolutionary history.

Coelacanths (Actinistia) are one of the most famous species-depauperate lineages. This clade diverged from other jawed vertebrates during the Silurian [3, 29, 30, 43] and represents the living sister clade to all other sarcopterygians, or lobe-finned fishes [3, 8, 55]. Despite their historical notoriety as a species-poor, morphologically conservative lineage, the fossil record of coelacanths has shown that this clade diversified into a wide variety of morphologies in the ancient past [17, 29, 49]. Fossil coelacanths show bursts of species diversity during the Devonian and Triassic [83] and achieved a high degree of body size variation ranging from diminutive species less than 5 cm long to 6 + m giants representing some of the largest freshwater fishes [18].

Only two species of coelacanths confined to deep ocean waters survive today: *Latimeria chalumnae* and *L. menadoensis* [39, 79]. These species diverged from other coelacanths during the Cretaceous [17, 83] and last share common ancestry over 35 million years ago [41]. There is also evidence for additional, deep splits among populations in the two recognized *Latimeria* species [44]. However, the secludedhabitats and small population sizes of extant coelacanths mean that there is a dearth of specimens available for assessing morphological variance in these populations. This precludes our ability to understand current coelacanth species diversity and morphological disparity, which might otherwise inform species delimitation in the fossil record.

The eastern margin of North America is known for its extensive fossil record from Triassic-Jurassic rift lakes that formed during the breakup of Pangaea (e.g., [65]. Several formations representing these ecosystems preserve the most extensive collection of coelacanths known, extant or extinct (e.g., [9, 70–72, 76]. Yet, just how many species of coelacanths lived in this region during the Triassic has remained contentious for over a century [9, 70–72, 76].

In this paper, we quantify phenotypic disparity and species richness in a sample of over 500 individual coelacanths from a single locality with a combined approach using tools from geometric morphometrics, meristics, and phylogenetics. This allows us to critically assess coelacanth diversity in the Triassic eastern North American rift, which leads us to recognize one new species and provides a basis for reanalyzing actinistian diversity in deep time. Our study reinforces the necessity of quantitative methods for species delimitation among depauperate fossil lineages and shows how assumptions about the species richness of a lineage might cause underestimation of their ancient diversity.

## Methods

### Sampling

In order to estimate the species richness of Triassic coelacanths in eastern North America, we examined over 500 specimens of coelacanths collected during the 1940s Firestone Library excavation in Princeton, New Jersey [70, 72]. Of these, n = 55 specimens possessed skulls with exceptional preservation allowing us to perform a variety of linear and geometric morphometric comparisons. We selected a subset of n = 19 individuals represented by articulated skulls and skeletons showing details of suspensorium, opercular series, and postcranial anatomy for Bayesian- and parsimony-based phylogenetic analyses. We also sampled an additional five specimens from the Old Granton Quarry in Bergen, New Jersey and examined a skull and partial skeleton of a large coelacanth collected in 1975 from the Solite Quarry site in North Carolina. Measurements made on this dataset using digital calipers were combined with measurement data from [29, 70, 72, 76]. Together, this dataset represents the largest known collection of coelacanth material from a single region and time (Carnian-Norian, e.g., [45]).

### Phylogenetic analysis

We conducted several rounds of phylogenetic analysis on the morphological dataset of Toriño et al. [83] with wildcard genera excluded, which consists of 48 taxa scored for 110 characters. To assess how different phylogenetic methodologies affected relationships among coelacanths, we conducted both Bayesian and parsimony analyses.

We conducted an analysis under parsimony using the program TNT v. 1.5 [34]. Initially, we performed a Wagner search with space for 1000 trees and default parameters for ratchet, tree fuse, drift, and sectorial search. This was followed by a round of traditional bisection-reconnection (TBR) branch swapping with space for 100,000 trees to explore additional topologies. The resulting MPTs were summarized in a strict consensus topology. We also resampled the dataset over 100 replicates to obtain bootstrap support values for branches. Parsimony analysis was conducted using both the dataset including YPM VPPU 14555 and without this specimen, which we resolved as a wildcard in the initial run. A list of apomorphies for each run is in the Additional file 2, and the inputted morphological matrix and output trees are included in the Additional file 3.

We conducted Bayesian analysis of the modified morphological dataset of Toriño et al. [83] and age dates for fossil occurrences taken from that study and additional sources for the new coelacanth material [45, 48] using the program BEAST 2.6.6 [6] with the fossilized birth–death (FBD) model as the tree prior [32]. A single uncorrelated lognormal clock was used with mean and standard deviation values of 1.0 and 0.33, respectively. We conducted three independent runs over 1 × 10^7^ million generations with a 1 × 10^6^ pre-burnin. We used Tracer v. 1.7.2 [67] to check for convergence of posteriors. The posterior set of trees generated from this analysis were summarized into a maximum clade credibility (MCC) topology using TreeAnnotator 2.6.4 [6] with a 25% burnin. The input xml file and resulting tree, state, and log files from the Bayesian analysis are included in the Additional file 3.

### Linear morphometric analyses

In order to assess simple dimensional differences among the sample of coelacanths examined in this contribution, we collected measurement data for the following dimensions: anteroposterior skull length from the tip of the premaxilla to the posterior end of the opercle, dorsoventral skull height from the base of the angular to the mid-length of the parietal, maximum orbit height and length, the number of ridges observed on the visible opercle of each specimen, and the number of angular foramina visible. The latter two counts were taken using light microscopy. We compared measurements for n = 55 of the best-preserved skulls from the Granton Quarry and Firestone sites, and then among these and an additional n = 7 specimens from these sites and other localities of the Newark Supergroup. Plotting was conducted in the R package ggplot2 [88].

### Species-site diversity and per-site size disparity

Based on the results of our phylogenetic, linear meristic and morphometric, and geometric morphometric analyses, we assembled catalogues of coelacanth species presence-absence data at several sites in eastern North America (Schainin 1943; Schaeffer [71, 72]; this study). Plotting was conducted in the R package ggplot2 [88]. As a metric of per-site size disparity, we calculated the difference in total length between the largest and smallest coelacanths reported from each of the localities we investigated. In several cases (i.e., Granton Quarry, Firestone Library), it was necessary to estimate the sizes of the largest reported individuals of the species †*Diplurus longicaudatus* based on complete specimens of the same species reported from elsewhere (i.e., YPM VP 630; Schaeffer [71]). Plotting was conducted in the R package ggplot2 [88].

## Results

### Geological and environment setting

The massive coelacanth collection presented here was found in the Firestone locality of Princeton, New Jersey during the excavation of the Firestone Library in 1946. This site is centered on the Newark basin, the largest of the exposed rift basins formed during the breakup of Pangea between the Late Triassic and Early Jurassic [53, 80]. Infilling of the basin occurred over approximately 30 million years in the Triassic and produced three main units in descending chronological order: the Stockton, Lockatong, and Passaic Formations [51, 53]. The Stockton Formation consists largely of red and purple clastic conglomerate rocks, red to yellow-grey well-sorted arkose, and red to brown siltstone and mudstone [19, 54]. Much of the great lateral extent of the geology in this formation has been interpreted as alluvial fans resulting from fluvial and lacustrine processes [80]. Conformably overlying the Stockton Formation is the Lockatong Formation. The Lockatong Formation covers an area of 7000 km^2^ and has a maximum thickness of approximately 1100 m [19]. The Lockatong Formation beneath the Firestone locality is around 450 m thick and dips 10 degrees north [19]. Sedimentary infilling of the Lockatong Formation reflects cyclical periods of the rise and fall of lakes, referred to as Van Houten cycles [61, 62, 85]. Van Houten cycles have a periodicity of approximately 20,000 years and are roughly divided into three sections chiefly containing large grey to red clastics to dolomites, laminated red to green organic-rich siltstone and claystone, and largely desiccated calcareous clastic units [19, 60]. These three sections are thought to correspond to periods of lake level rise, lake level stasis, and lake level fall, respectively, driven by orbital climate dynamics [51, 80]. Lacustrine ecosystem changes composed of Van Houten cycles ranging from ~ 90 kyr to ~ 2000 kyr have also been observed in the Lockatong Formation [51, 53, 60].

The Firestone locality lies approximately 70 m above the contact between the Stockton and Lockatong Formations [74]. The collection of coelacanths examined for this study were all preserved within a restricted, < 20 cm layer of argillite. A number of specimens are preserved in regions where fractionation occurred along bedding planes, resulting in bands of argillite-derived soft limnotic clay [74]. Fossils preserved in these regions are far more visible and better preserved than those found in the unchanged argillite. Other fishes found in surrounding regions of the Lockatong Formation include the actinopterygians †*Turseodus*, †*Cionichthys*, and †*Synorichthys* and the shark †*Carinacanthus* [59, 63].

Coelacanth fossils have also been found in surrounding regions of the Lockatong and Stockton Formations. Shainin [76] described a collection of †*Diplurus* from the Granton quarry in North Bergen, New Jersey. The Stockton Formation at this locality is approximately 700 m thick; †*Diplurus* is found in the upper section, approximately 640 m above the base [74]. The lithology of this section consists of alternating layers of sandy to silty sandstone and dark sandy to argillaceous shales [76]. Small coelacanths are found here in the dark shale layers [76]. Similar assemblages have been found embedded in dark shale in the upper half of the Lockatong Formation in Montgomery County, Pennsylvania.

Boonton, New Jersey is the site of one of specimens shown as part of the collection here. The Boonton Formation consists of large red siltstone and sandstone sections alternating with grey siltstone, as well as red, brown and grey clastics, and evaporite layers [61, 62]. The unit is part of the Passaic Group (formerly the Brunswick Formation) and is among the youngest sedimentary units in the Newark Basin [61, 62]. Myriad other fish fossils, including †*Semionotus*, †*Redfieldius*, †*Dictyopyge,* and †*Ptycholepis*, have been found in the uppermost section, which is composed of a grey siltstone laminite [61, 62, 73]. Single specimens have also been found in the Lockatong and Passaic formations in the Danville area and Fauquier County, Virginia.

Various remains of †*Diplurus longicaudatus* have also been identified in the Connecticut Valley. While small coelacanth specimens assigned to †*Diplurus* are the vast majority of coelacanths present in the New York-New Jersey areas, such as the Firestone Library site, †*D. longicaudatus* is the only species to have been found in the Connecticut Valley [74]. The lithology of the Shuttle Meadow Formation, which bears †*Diplurus* in this region, consists largely of arkose, sandstone, small amounts of shale, and siltstone [74].

Other samples analyzed in this paper were collected from the Solite Quarry in North Carolina-Virginia, USA. This section rests in the Dan River-Danville basin, a half-graben along the Chatham fault zone of the Mesozoic rift system [5, 64]. This region consists largely of lacustrine shales, sandstones, and mudstones that were layered cyclically and are fossiliferous [28, 64]. Over 30 cyclical layers are present in this area, which are thought to reflect Milankovitch Cycles (orbital dynamic-driven cycles in lake-depth similar to Van Houten Cycles) and contain some of the most productive Triassic fossil assemblages in the world [5, 28].

### Phylogenetic analyses

Phylogenetic analysis of the specimen-level dataset (modified from [83]) under Bayesian and parsimony frameworks produced similar positions for the sampled coelacanth specimens (Figs. 2, 3).

**Fig. 1:**
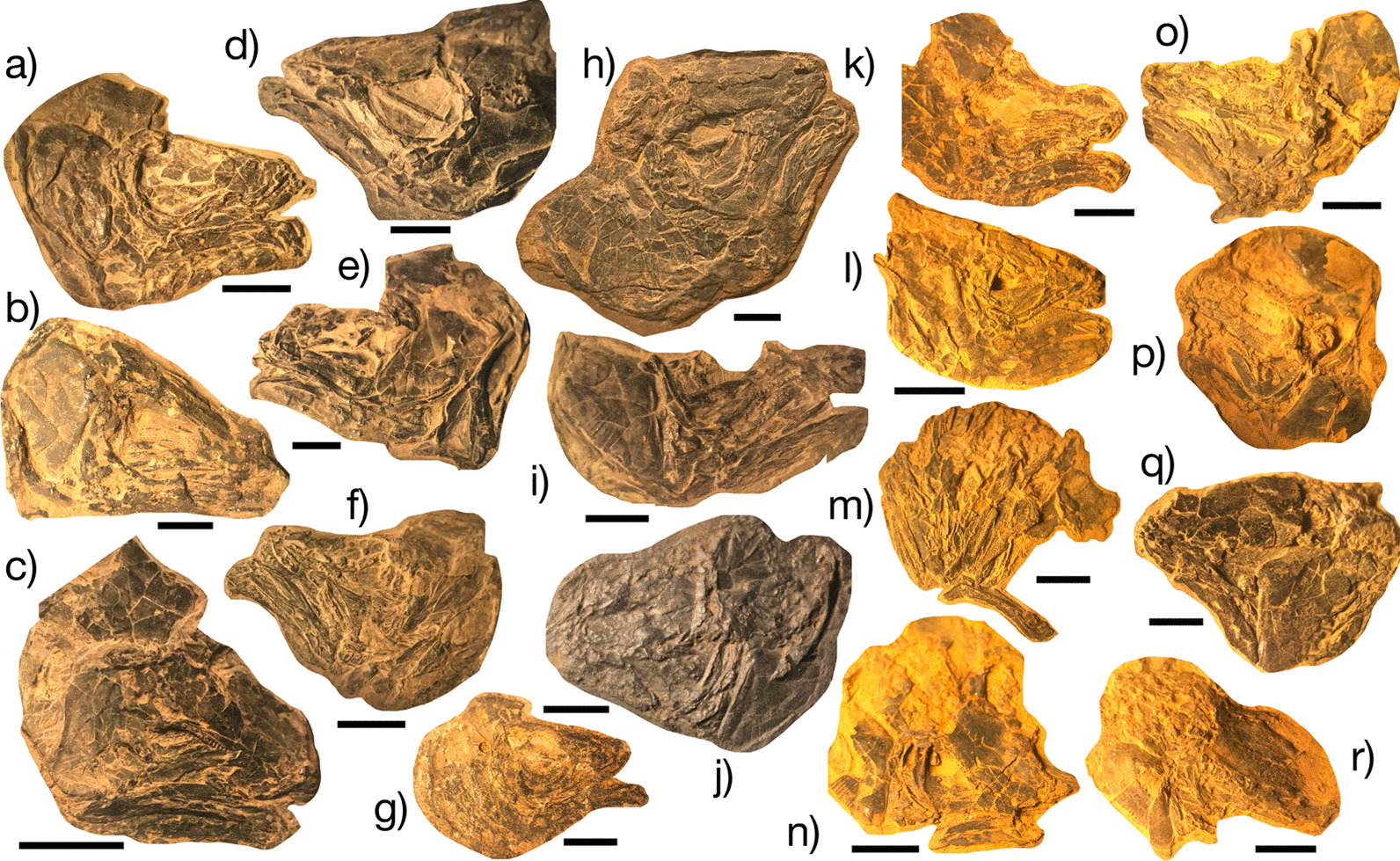
Examples of exceptionally preserved †*Diplurus* from the Firestone Library Excavation and Granton Quarry sites. **a** YPM VPPU 14,944 †*D. newarki*, **b** YPM VPPU uncatalogued †*D. newarki*, **c** YPM VPPU 14918a †*D. newarki,* **d** YPM VPPU 14,558 †*D. newarki*, **e** YPM VPPU uncatalogued †*D. newarki*, **f** YPM VPPU 14,924 †*D. enigmaticus*, **g** YPM VPPU uncatalogued †*D. newarki*, **h** YPM VPPU 14,920 †*D. newarki*, **i** YPM VPPU uncatalogued †*D. newarki*, **j** YPM VPPU 14,929 †*D. newarki*, **k** YPM VPPU 29,366 †*D. newarki*, **l** YPM VPPU 14,933 †*D. newarki*, **m** YPM VPPU 14,935 †*D. newarki*, **n** YPM VPPU 14,949 †*D. enigmaticus*, **o** YPM VPPU 14,932 †*D. newarki*, **p** YPM VPPU 14,940 †*D. newarki*, **q** YPM VPPU 14,921 †*D. newarki*, and **r** YPM VPPU 14,939 †*D. enigmaticus*

**Fig. 2:**
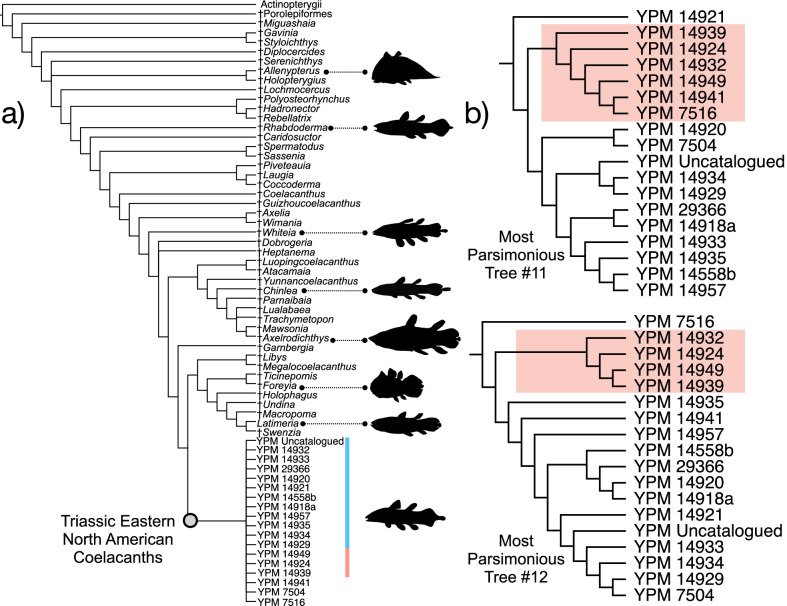
Parsimony phylogenetic hypothesis of the exceptional eastern North American coelacanth sample. **a** Strict consensus topology resulting from the parsimony analysis with YPM VPPU 14,955 removed, with **b** equally most parsimonious trees showing recovery of a clade with striated opercles (highlighted to match color in Fig. 1). Silhouettes of coelacanths (except *Latimeria* and †*Foreyia*) drawn after Schaeffer [11, 18, 27, 74]

Parsimony analysis of the dataset (Fig. 2; Additional file 1: Fig. S1) finds largely unresolved relationships among actinistians and places the Firestone Library and Old Granton Quarry coelacanths in a polytomy at the base of this lineage in the strict consensus topology of 36 most parsimonious trees (MPTs) found with moderate support (bootstrap value = 0.5). MPTs produced from this analysis (length = 348; consistency index = 0.356, retention index = 0.732) position the eastern North American coelacanth clade (bootstrap support = 5) sister to Latimeriidae (bootstrap support = 6) and resolve distinct subgroups within the eastern North American lineage delimited by the ornamentation of the opercle (Fig. 2). The uncertainty in the phylogenetic relationships among the eastern North American coelacanth clade, which drives the production of the polytomy in the consensus tree, is likely attributable to the lack of material known for YPM VPPU 14,555, a Firestone Library specimen referred to †*Diplurus longicaudatus.* Exclusion of this partial skull resulted in the resolution of a monophyletic eastern North American coelacanth group positioned as the sister to the Latimeriidae in all 18 MPTs (length = 347, consistency index = 0.355, retention index = 0.731) found, as well as in the strict consensus topology (Fig. 1a). 12 MPTS show the formation of a clade of eastern North American Triassic coelacanths sharing a striated opercle (Fig. 2b). The monophyly of Triassic eastern North American coelacanths is supported by a low bootstrap value of 34 (Additional file 1: Fig. S1d), and a value of 0 supported the position of Triassic eastern North American coelacanths in the Latimeriidae.

**Fig. 3:**
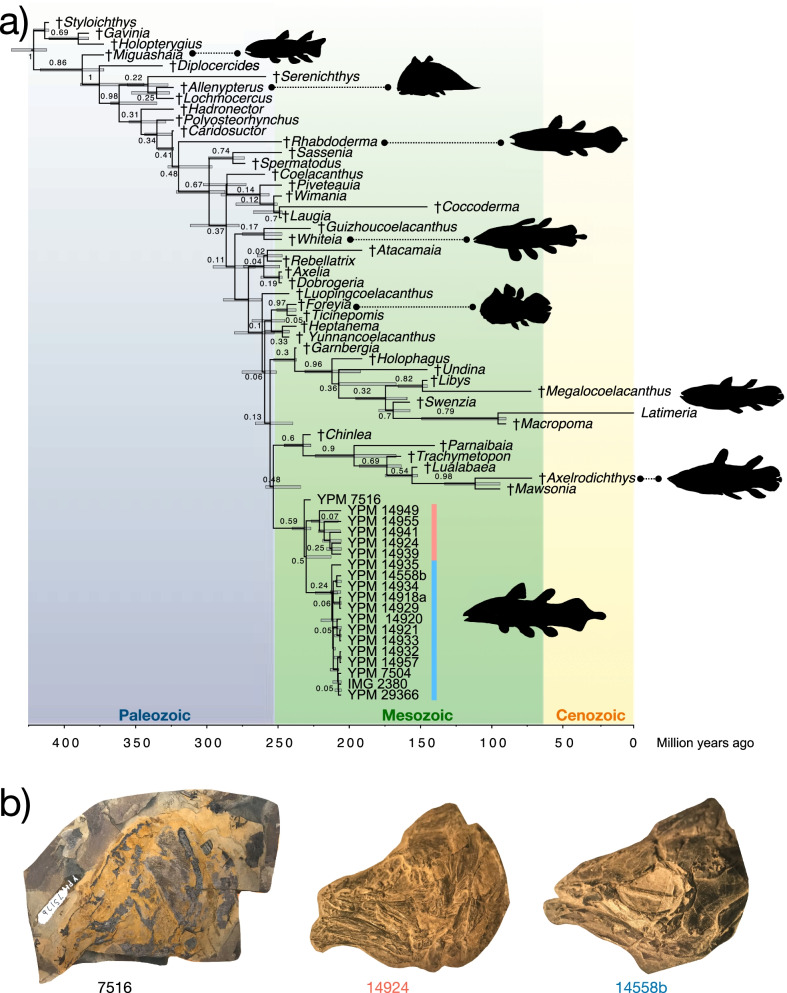
Bayesian phylogenetic hypothesis of the exceptional eastern North American coelacanth sample. **a** Bayesian time-calibrated maximum clade credibility tree of coelacanth relationships, including specimen-level analysis of Lockatong specimens. The clade identifiable as †*Diplurus newarki* is highlighted in blue and the new species of †*Diplurus*, †*D. enigmaticus*, highlighted in red. **b** Exemplar skulls of three major lineages within †*Diplurus*, color-coded to match the phylogeny (see **b**)

In the Bayesian time-calibrated tree (Fig. 3), all eastern North American species are resolved as a monophyletic lineage within †Mawsoniidae, a cosmopolitan Mesozoic coelacanth clade [4, 14–16, 25, 26, 50, 81, 83], as the sister lineage to all Jurassic-Cretaceous mawsoniids included in the dataset. The monophyly of eastern North American Triassic coelacanths is supported by a moderate posterior value of 0.58. The inclusion of these eastern North American coelacanths in †Mawsoniidae (excluding †*Heptanema* and †*Yunnancoelacanthus*) is supported by a higher posterior value of 0.76. The eastern North American clade is estimated to diverge from other mawsoniids 253.22 million years ago (95% CI: 234.10–258.81 Ma), approximately the age of the Permian mass extinction.

The eastern North American clade itself is divided into four distinct groups. The first to diverge consists of the large-bodied specimen YPM VP 7516 from the Carnian of North Carolina preliminarily referred to †*Diplurus longicaudatus* in the Yale Peabody Museum collections (Fig. 3). This result seems to be primarily driven by the age of YPM VP 7516. Next, two clades consisting of coelacanths from the Lockatong Formation diverge from each other separated by the presence of extensive opercle ornamentation. Two subclades of note are present in the clade consisting of species with extensively ornamented opercles. One of these subclades consists of the large Firestone coelacanth specimen YPM VPPU 14555 (cf. †*Diplurus longicaudatus*) and the small skull YPM VPPU 14941 (Fig. 3). The other subclade consists of all small-bodied coelacanth specimens from the Firestone Library excavation with heavily striated opercles (Fig. 3). The striated opercle lineage and that containing YPM VPPU 14555 and YPM VPPU 14941 form the sister clade to all coelacanth with minimal opercle ornamentation from Firestone and Old Granton Quarry (Fig. 3).

### Morphometric analyses

**Fig. 4:**
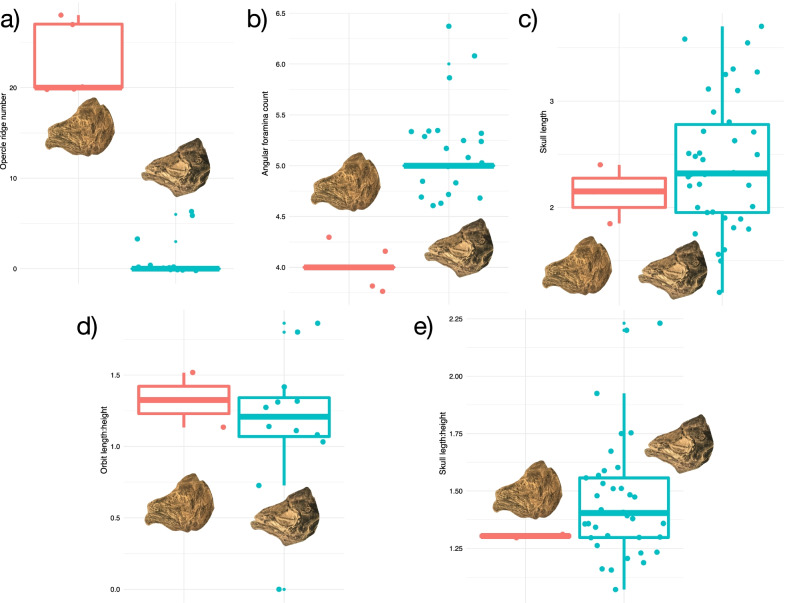
Linear morphometrics and counts of the Lockatong Formation coelacanth sample. Comparative graphs showing differences in cranial features (**a**, **b**), skull dimensions (in cm) **c** and skull proportions **d**, **e** among †*Diplurus newarki* and †*Diplurus enigmaticus* sp. nov. Red indicates skulls assigned to †*Diplurus enigmaticus* sp. nov. Blue indicates skulls assigned to †*Diplurus newarki* sp. nov

**Fig. 5:**
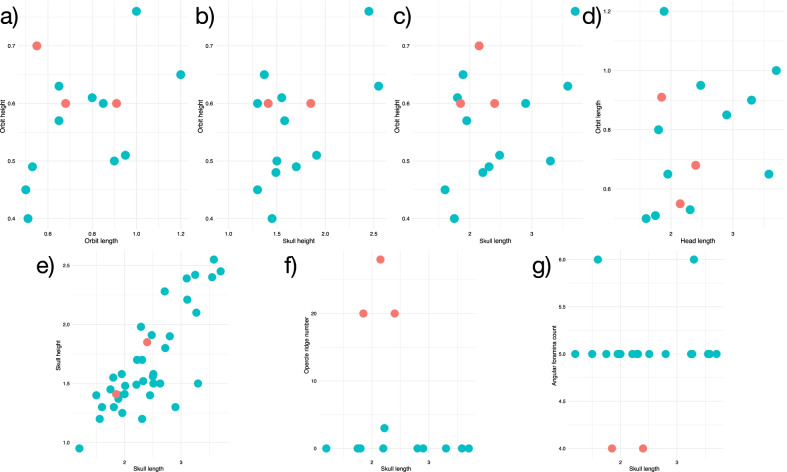
Comparative proportions of Firestone coelacanth skulls. Plots showing associations between **a**–**e** different orbit and skull measurements (in cm) figured and **f**, **h** the absence of association between **g** opercle ridge count, **g** angular foramina count, and skull length (which roughly approximates size). Red indicates skulls assigned to †*Diplurus enigmaticus* sp. nov. Blue indicates skulls assigned to †*Diplurus newarki* sp. nov

To investigate the morphological variation in the eastern North American coelacanth sample that might underlie our phylogenetic results, we performed both linear and 2D geometric morphometric analyses on the sample of coelacanths from the Firestone Library locality (with the exceptionally preserved Old Granton Quarry specimen YPM VPPU 14558a also included) to assess the level of variation in skull proportions, ornamentation, and neurovasculature in this sympatric population or set of populations (Figs. 4, 5). Two groups consistently distinguished by the frequency of radiating striations on the opercle (0–6 vs. 20 +) and foramina on the angular (5 + vs. 4) were found to exist in the Firestone sample of small-bodied coelacanths (Fig. 4a, b). These different groups differed little in size and showed similar skull and orbital sizes (Fig. 4c, e) and proportions (Fig. 5). Higher opercle striation and angular foramina counts are not associated with longer skull or deeper skulls in the Firestone Library and Granton Quarry sample (Fig. 5), suggesting these traits are independent of head and body dimensions.

We expanded our linear morphometric dataset to include records of coelacanths from several other localities across eastern North America, including further samples from the Old Granton Quarry site, the giant North Carolina specimen YPM VP 7516, several specimens of †*Diplurus longicaudatus*, skulls assigned by Schainin (1943) to the species †*Osteopleurus milleri*, and an opercle referred by [76] to †*O. milleri grantonensis*. We found that cranial material assigned to †*O. m. milleri* falls within the range of variation seen in the crania of †*D. newarki*, suggesting these are synonymous [74]. Skulls assigned to †*D. longicaudatus* (including YPM VP 7516) were much larger than all other specimens and possessed the numerous opercle ornamentations characteristic of that species [74]. Finally, the opercle from Granton Quarry tentatively referred to †*O. milleri grantonensis* by Schainin (1943) shows a similar degree of ornamentation as the ornamented-opercle group from Firestone Library and belonged to a small-bodied coelacanth of similar size.

### Systematics

Actinistia Cope 1871.

Latimerioidei Schultze, 1993.

†*Diplurus* Newberry 1878.

†*Diplurus enigmaticus* sp. nov.

**Material.** YPM VPPU 14924 (holotype), skull and mandibles (Firestone Library). YPM VPPU 14949, 14939,14943, 14558b; skulls with mandibles (Firestone Library). AMNH 15222, opercle (Granton Quarry).

**Diagnosis.** †*Diplurus enigmaticus* is distinguished by the following combination of features: maximum standard length of approximately 150 mm (shared with †*Diplurus newarki*; 690 + mm in †*D. longicaudatus*), numerous (> 20) well-delimited radiating ridges on opercle (maximum of seven observed in †*D. newarki*; irregular lineations and tubercles present in †*Diplurus longicaudatus*; Figs. 4, 5); four angular foramina (zero to two in †*D. longicaudatus*; five or more in †*D. newarki*; Figs. 4, 5); premaxilla with reduced number (8) of enlarged, conical teeth (11 in each element in †*D. newarki*; [74])..

**Remarks**. Schaeffer [7, 70, 71] provided comprehensive descriptions of the Lockatong, Stockton, and Boonton Formation †*Diplurus* material, including several specimens examined for this study. We refer the reader to these illustrated osteologies for details about the anatomy of the genus. The differential diagnosis given by Schaeffer [74] for †*Diplurus* (†*D. longicaudatus* + †*D. newarki)* includes the following features: (1) incomplete braincase ossification; (2) three posterior flanges on the anterior ethmoid; (3) large ovoid antotic flanges on the basisphenoid; (4) ossified otooccipital region; (5) basisphenoid not fused to parasphenoid; (6) ungrouped parasphenoid teeth; (7) largely unornamented skull dermal bones; (8) numerous small rostral bones; (9) small tooth-bearing premaxilla; (10) three subequal frontal-ethmoid shield bones; (11) anterior frontals meet at midline (variable; (12) rectangular supratemporals do not reach posterior to intertemporals; (13) large dermosphenotic medially borders intertemporal; (14) extrascapulars reduced in size; (15) absence of distinct antorbital; (16) no sclerotic ring; (17) large sensory canal pores line postorbital and squamosal; (18) absence of subopercle; (19) short lower jaw; (20) dentary and splenial elongated; (21) lower jaw concave. Of these, 1, 2, 3, 4, 5, 6, 8, 9, 10, 11, 12, 13, 14, 15, 16, 18, 19, and 21 are found together in other Triassic or post-Devonian coelacanths described subsequently (i.e., *Foreyia* and *Ticinepomis*; [17, 69, 74]). Specimens of †*Diplurus enigmaticus* show features 7, 8, 9, 11, 12, 13, 14, 15, 16, 17, 18, 19, 20, and 21 and have previously been assigned to this genus [74]. Apart from the features noted in the diagnosis section, †*Diplurus enigmaticus* individuals fall within the range of variation observed in †*Diplurus newarki* and †*Diplurus longicaudatus*. Accordingly, our comparisons below focus on the three features that we argue are apomorphies of †*Diplurus enigmaticus*.

**Fig. 6:**
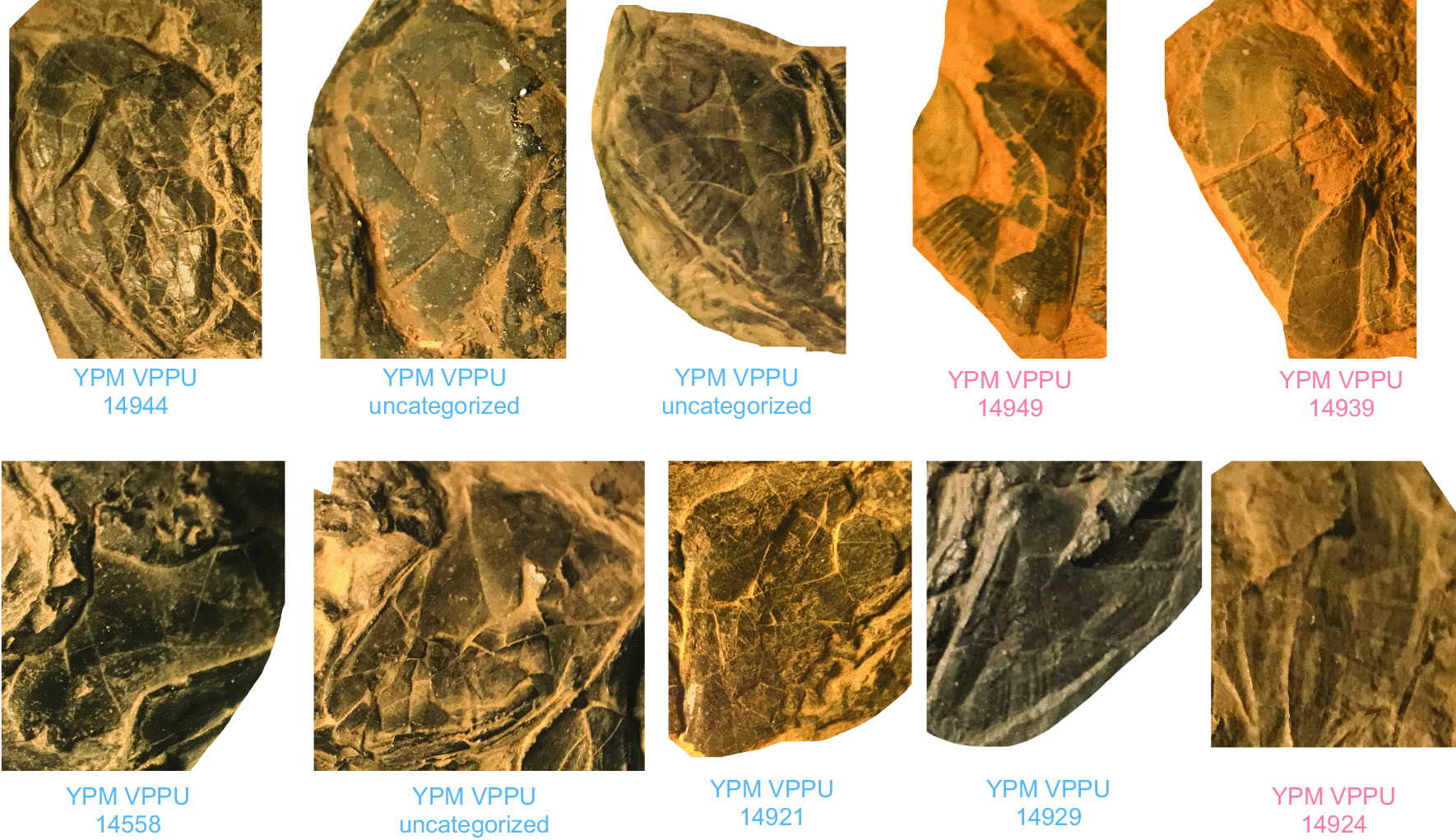
Comparative opercle anatomy of †*Diplurus newarki* and †*Diplurus enigmaticus* sp. nov. Blue denotes specimens of †*Diplurus newarki*, red denotes specimens of †*Diplurus enigmaticus*

**Fig. 7:**
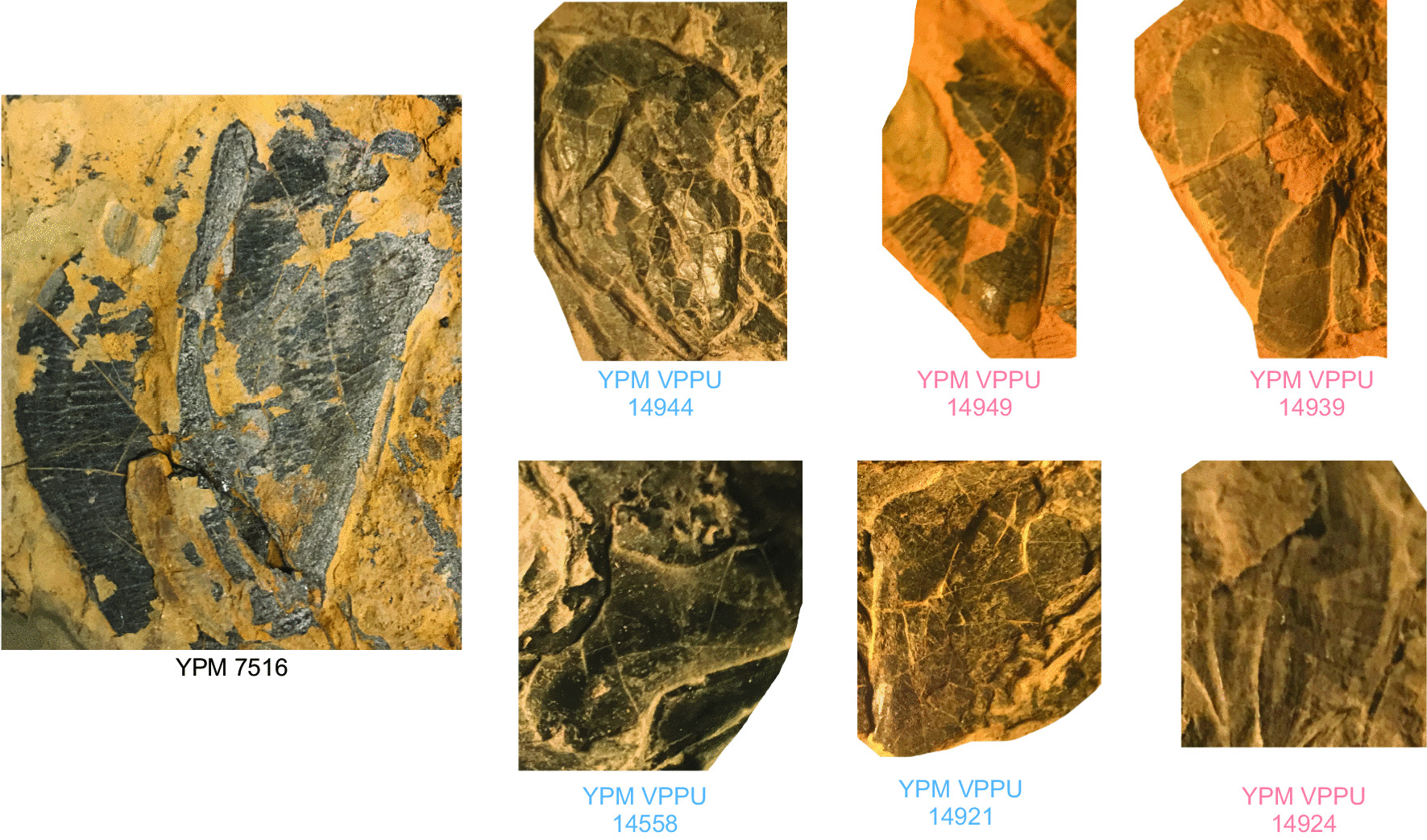
Comparative opercle anatomy of †*Diplurus newarki* and †*Diplurus enigmaticus* sp. nov. Black denotes specimens of †*Diplurus longicaudatus*, blue denotes specimens of †*Diplurus newarki*, red denotes specimens of †*Diplurus enigmaticus*

**Fig. 8:**
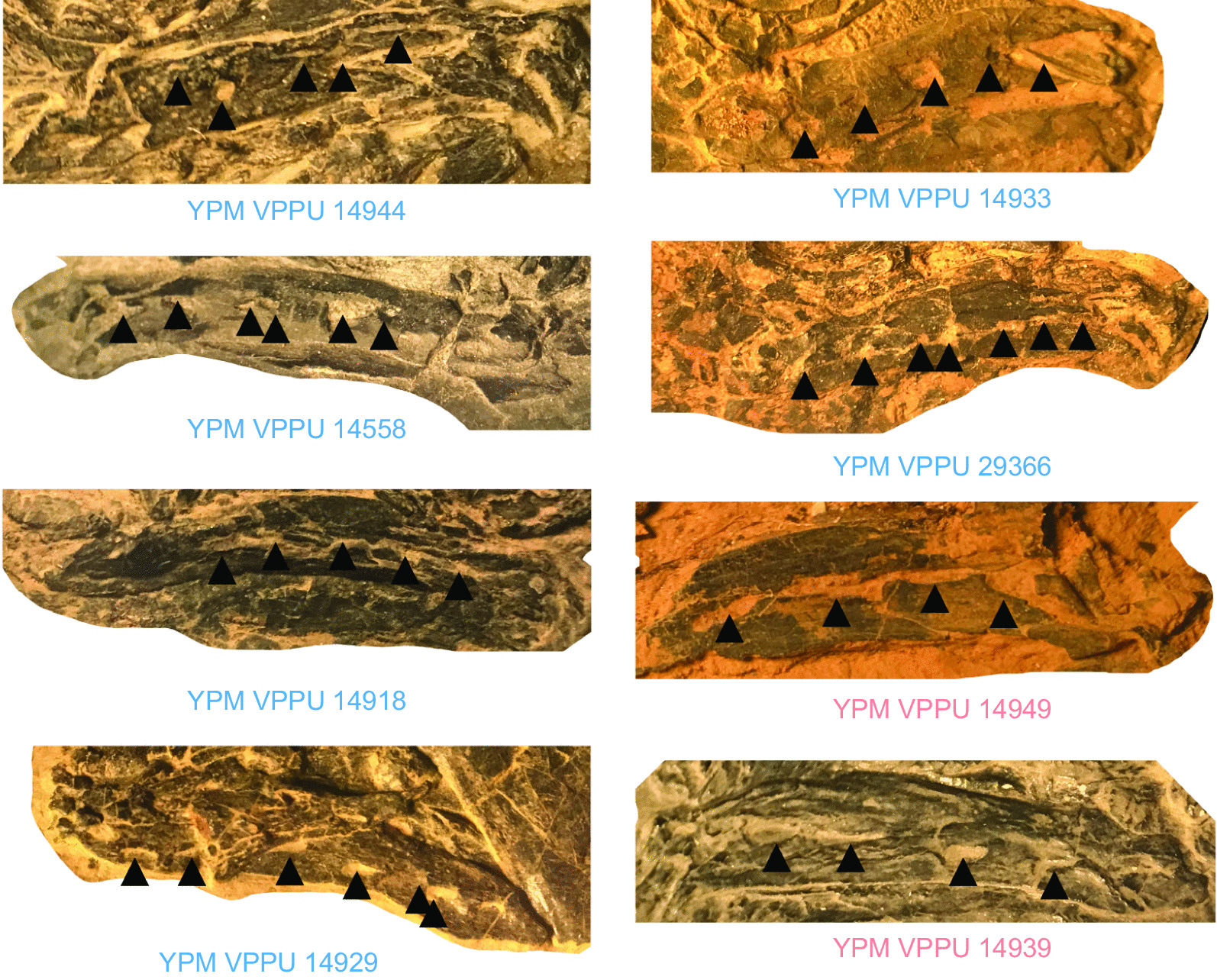
Comparative angular anatomy of †*Diplurus newarki* and †*Diplurus enigmaticus* sp. nov. Blue denotes specimens of †*Diplurus newarki*, red denotes specimens of †*Diplurus enigmaticus*

Schainin (1943) considered the presence of discrete striations on the opercle to be a diagnostic apomorphy of †*Osteopleurus*, whereas Schaeffer [74] suggested this feature was not diagnostic based on the variation in opercle ornamentation he observed in the Firestone Library excavation coelacanth assemblage. Our quantification of key skull characteristics in coelacanths from both Firestone Library and Granton Quarry shows that small-bodied coelacanths with heavily striated opercles also consistently possess four angular foramina, whereas †*Diplurus newarki* consistently possesses fewer than 10 opercle striations and five angular foramina. There is no continuous variation in either of these features. The opercle ornamentation of †*D. enigmaticus* also distinguishes this species from the much larger †*D. longicaudatus.* The ornamentation on the opercle of †*D. longicaudatus* consists of numerous weak ridges that span the anteroposterior axis of the opercle and run posteroventrally (Fig. 8). In †*D. enigmaticus*, these ridges are straightened and radiate from a center located midway along the dorsoventral axis of the bone (Figs. 6, 7, Fig. 8). Further, there is no evidence for more than one or two distinct angular foramina in any specimen of †*D. longicaudatus* [71, 72] in contrast to the four foramina found in †*D. enigmaticus.* †*D. enigmaticus* almost certainly does not represent a juvenile form of †*D. longicaudatus*, as the skull and skeleton are strongly ossified and are not drastically proportionally different [71] as in the skulls of small juveniles and adults of the extant coelacanth *Latimeria* [24].

**Fig. 9:**
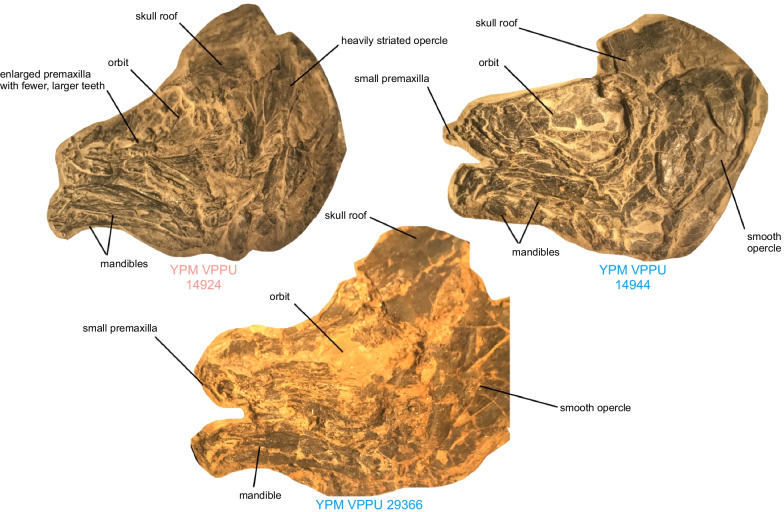
Comparative cranial anatomy of †*Diplurus newarki* and †*Diplurus enigmaticus* sp. nov. Blue denotes specimens of †*Diplurus newarki*, red denotes specimens of †*Diplurus enigmaticus*

A third osteological feature that distinguishes specimens of †*D. enigmaticus* from †*D. newarki* is the size of the premaxillary dentition (Fig. 9). YPM 14924, the holotype complete skull of †*D. enigmaticus*, includes an enlarged premaxilla with at least 8 conical teeth that appear much larger than in specimens of †*D. newarki*, such as YPM 14558a (see also Fig. 4 in [74]). Specimens of †*D. newarki* also possess a higher premaxillary tooth count of 11 tooth positions in each premaxilla [74].

## Discussion

### Diversity and phylogenetic position of †*Diplurus*

In this study, we have quantified coelacanth diversity at several exceptional assemblages from the Triassic rift lakes of eastern North America. This approach allows us to quantitatively assess the validity of several previously named species from this region [76] and provide strong phylogenetic and morphometric evidence for the existence of the unrecognized, small-bodied form, †*Diplurus enigmaticus*, living in sympatry with the similarly-sized †*Diplurus newarki* and the much larger †*Diplurus longicaudatus* at the Firestone Library excavation and possibly Granton Quarry [70–72]. Our results also support synonymy of †*Osteopleurus milleri* with †*D. newarki* [74] and corroborate the hypothesis that minor size differences among coelacanth specimens from northeastern North America do not warrant the recognition of new species (thereby making †*O. m. grantonensis* a nomen dubium sensu [74].

Intensive sampling of these Triassic eastern North American coelacanths also provides new information on the evolutionary relationships of these freshwater species. Placing †*Diplurus* among coelacanths has been difficult. Alternative approaches to phylogenetic reconstruction have allied this genus with both the Latimeriidae and the extinct coelacanth clade †Mawsoniidae, which was the dominant lineage during much of the Mesozoic [15, 17, 18, 83]. Although we still found conflicts between the relationships of †*Diplurus newarki*, †*Diplurus longicaudatus*, and †*D. enigmaticus* to other coelacanths resolved in parsimony and Bayesian frameworks (cf. [83], our intensive sampling suggests (1) that these eastern forms are more likely early-diverging members of †Mawsoniidae than Latimeriidae (Figs. 2, 3,[17]) and (2) the somewhat ambiguous phylogenetic positions of these eastern North American coelacanth species may result from an incomplete understanding of character evolution at a critical junction in the coelacanth tree: the divergence of the Mesozoic mawsoniids from *Latimeria* and its closest relatives in Latimeriidae [13, 15, 17, 83].

### Coelacanths as a depauperate vertebrate clade

The existence of depauperate, evolutionarily stagnant lineages has been a matter of great interest since this pattern was first recognized (e.g., [12, 13, 21, 47, 82]. One central point of contention has been whether continuously low species diversity across time scales of tens of millions of years in many of these clades is driven by genuinely low speciation rates or simply the incompleteness of the fossil record (e.g., [12, 13, 18, 57, 77]. In the case of depauperons, rigorous species delimitation is therefore all the more essential, as these clades might show a lower level of morphological disparity across species boundaries if taxic depauperacy is coupled with reduced evolvability. Extant coelacanths present an example of this phenomenon,despite diverging over 30 million years ago, the two recognized species of *Latimeria* vary little in morphology [39, 41, 79]. The low number of characteristics distinguishing coelacanths with old common ancestors obscures whether deep divergences in this clade are indicative of speciation [44].

The diversity and size disparity of coelacanths found in the Triassic of eastern North America is higher than most previously reported assemblages of actinistians from the Mesozoic. Although multiple coelacanths have been described from the same geological units dating to the early Mesozoic [17], our site-based approach confirms that three species spanning a large range of body sizes were living in the same lakes and waterways. At the same time, the anatomy of all three species at Firestone and Granton Quarry are remarkably similar,these species all show similar fusiform body plans and differ extensively only in the ornamentation of their opercles, number of foramina in their lower jaws, the size and number of their premaxillary teeth, the form of their scalation, and the form and counts of their fins [71, 72] The observation of high size disparity coupled with low skeletal differentiation in the Lockatong coelacanth fauna supports the observation that the prevailing pattern in this clade is morphological conservatism [13, 15, 18, 83, 87],but see [17].

The species diversity of †*Diplurus* observed in Lockatong and Boonton Formation assemblages clearly contrasts with the view of coelacanths as a perpetually depauperate lineage (e.g., [13, 18, 52]). Instead, our results underscore the importance of quantitative approaches to species delimitation in the fossil record (e.g., [84]). In the case of coelacanths, our understanding of the evolutionary history of the total clade might be warped by observations of the evolutionary history of the crown group. *Latimeria* is currently represented by two species with an estimated common ancestor living > 30 million years ago [41, 46] that may have consistently lived in the marine benthos [20]. Further, *Latimeria chalumnae* and *L. menadoensis* possess slow molecular substitution rates at selectively constrained genes than most vertebrates (e.g., [3, 7, 58, 86]), although other regions of the genome thought to undergo neutral evolution (i.e., fourfold degenerate sites) show similar rates of change to other chordates [56].

There is genomic evidence for additional deep (> 10 million year) divergences among extant coelacanth populations that may imply unsampled extant coelacanth diversity [44, 58]. However, the deep-marine ecology, restricted distribution, and low populations of extant *Latimeria* greatly restrict our ability to investigate phenotypic disparity in the only extant actinistians [44]. Our analyses, which examine the largest morphological dataset for sympatric coelacanth individuals available, demonstrate how phenotypic variability denoting probable species distinctions might be overlooked even in sympatric populations of similarly-sized species (e.g., †*Diplurus newarki* and †*D. enigmaticus*). Thus, the depauperacy of coelacanths and the extensive temporal ranges of several mawsoniid and latimeriid genera might be artifacts of unrecognized subtle species distinctions in this species-poor clade rather than reflecting the existence of exceptionally long-lived genera [18, 27]. In contrast to what might be expected based on the apparent low morphological disparity and genomic rates of change in the crown group, the species richness of coelacanths and other depauperons may still largely be hidden in the geological past.

## Supplementary Information

**Additional file 1.**Apomorphies optimized in phylogenetic analyses. Additional Figure Captions.

**Additional file 2.** Measurement data.

**Additional file 3.** Phylogenetic data.

## Data Availability

All data is in the manuscript and the supplement. All material examined is in the collections of the Yale Peabody Museum of Natural History, a public repository in New Haven, CT. No living animals were examined.
