# Correction: Species delimitation and coexistence in an ancient, depauperate vertebrate clade

**DOI:** 10.1186/s12862-022-02053-2

**Published:** 2022-08-11

**Authors:** Chase Doran Brownstein, Immanuel Chas Bissell

**Affiliations:** 1 Stamford Museum and Nature Center, Stamford, CT USA; 2 Department of Ecology and Evolutionary Biology, Yale University, New Haven, CT USA; 3 Department of Earth and Planetary Sciences, Yale University, New Haven, CT USA

Correction: BMC Ecology and Evolution (2022) 22:90 10.1186/s12862-022-02043-4.

Following the publication of the original article [1], we were notified that the Zoobank LSID was not included. After the line "†Diplurus enigmaticus sp. nov." in the taxonomy section, the below needs to be inserted:

- **LSID** urn:lsid:zoobank.org:pub:F29E460E-D072-4C3B-912C-928D8677CC2C.

The original article [1] has been corrected.
