# Supplementary material for: Species delimitation and coexistence in an ancient, depauperate vertebrate clade
Source: BMC Ecol Evol. 2022 Jul 12;22:90. doi: 10.1186/s12862-022-02043-4 (PMC9277872; doi:10.1186/s12862-022-02043-4)
Supplement: Supplementary file 1 — Additional file 1.Apomorphies optimized in phylogenetic analyses. Additional Figure Captions. [file 12862_2022_2043_MOESM1_ESM.docx]

**Supplement To: Species delimitation and coexistence in an ancient, depauperate vertebrate clade**

**Table of Contents.**

1. **Apomorphies optimized in phylogenetic analyses.**
2. **Supplementary Figure Captions.**

**I. Apomorphies optimized in phylogenetic analyses.**

Note: all apomorphies are given in TNT format, i.e. character 0 below is the first character in the matrix.

with *D. longicaudatus* YPM VPPU 14555 from Firestone:

CI: 0.356

TL: 348 steps

RI: 0.732

Total MPTS: 36

Tree 36 :

    Actinopterygii :

      No autapomorphies

    Porolepiformes :

      Char. 11: 1 --> 0

      Char. 66: 0 --> 1

      Char. 79: 0 --> 1

*Diplocercides* :

      Char. 79: 0 --> 1

      Char. 82: 0 --> 1

*Rhabdoderma* :

      Char. 45: 0 --> 1

      Char. 76: 1 --> 0

      Char. 77: 1 --> 0

*Caridosuctor* :

      No autapomorphies

*Hadronector* :

      No autapomorphies

*Rebellatrix* :

      No autapomorphies

*Polyosteorhynchus* :

      Char. 20: 0 --> 1

      Char. 96: 0 --> 1

*Allenypterus* :

      Char. 58: 0 --> 1

*Lochmocercus* :

      Char. 20: 0 --> 1

*Coelacanthus* :

      Char. 3: 0 --> 1

      Char. 9: 1 --> 0

      Char. 25: 0 --> 1

      Char. 36: 0 --> 1

      Char. 41: 0 --> 1

      Char. 46: 0 --> 1

*Spermatodus* :

      Char. 14: 0 --> 1

      Char. 25: 0 --> 1

      Char. 69: 1 --> 0

*Whiteia* :

      Char. 4: 1 --> 0

      Char. 43: 0 --> 1

      Char. 56: 0 --> 1

      Char. 83: 0 --> 1

      Char. 104: 0 --> 1

*Laugia* :

      Char. 6: 1 --> 0

      Char. 25: 0 --> 1

      Char. 30: 1 --> 0

      Char. 55: 1 --> 0

*Sassenia* :

     Char. 68: 1 --> 0

*Chinlea* :

     Char. 55: 0 --> 1

     Char. 66: 0 --> 1

     Char. 97: 1 --> 0

     Char. 105: 0 --> 1

*Holophagus* :

     Char. 0: 0 --> 1

     Char. 98: 0 --> 1

*Undina* :

     Char. 4: 1 --> 0

     Char. 62: 1 --> 0

*Coccoderma* :

     Char. 22: 0 --> 2

     Char. 26: 0 --> 1

     Char. 36: 0 --> 1

     Char. 37: 0 --> 1

     Char. 48: 1 --> 0

     Char. 88: 0 --> 1

     Char. 96: 1 --> 0

     Char. 105: 0 --> 1

*Libys* :

     No autapomorphies

*Mawsonia* :

     Char. 16: 0 --> 3

     Char. 34: 1 --> 0

     Char. 70: 0 --> 1

*Macropoma* :

     Char. 1: 0 --> 2

     Char. 66: 1 --> 0

     Char. 88: 0 --> 1

     Char. 103: 0 --> 1

*Latimeria* :

     Char. 3: 0 --> 1

     Char. 45: 0 --> 1

     Char. 49: 0 --> 1

     Char. 106: 1 --> 0

*Miguashaia* :

     Char. 65: 0 --> 1

*Axelrodichthys* :

     Char. 13: 0 --> 1

     Char. 19: 1 --> 0

     Char. 37: 0 --> 1

*Holopterygius* :

     Char. 92: 1 --> 0

     Char. 98: 0 --> 1

*Garnbergia* :

     Char. 35: 0 --> 1

     Char. 41: 1 --> 0

     Char. 48: 0 --> 1

     Char. 97: 1 --> 0

     Char. 103: 1 --> 0

*Parnaibaia* :

     Char. 2: 0 --> 1

     Char. 3: 0 --> 1

     Char. 25: 1 --> 0

     Char. 77: 0 --> 1

*Swenzia* :

     Char. 1: 0 --> 1

*Guizhoucoelacanthus* :

     Char. 24: 1 --> 0

     Char. 42: 0 --> 1

     Char. 51: 1 --> 0

     Char. 94: 1 --> 0

     Char. 105: 0 --> 1

*Piveteauia* :

     Char. 12: 0 --> 1

     Char. 21: 0 --> 1

     Char. 52: 0 --> 1

     Char. 78: 0 --> 1

*Axelia* :

     Char. 67: 0 --> 1

*Wimania* :

     No autapomorphies

*Megalocoelacanthus* :

     Char. 4: 1 --> 0

*Luopingcoelacanthus* :

     Char. 34: 1 --> 0

     Char. 41: 1 --> 0

*Yunnancoelacanthus* :

     Char. 7: 1 --> 0

     Char. 8: 1 --> 0

     Char. 9: 0 --> 1

     Char. 13: 0 --> 1

     Char. 14: 1 --> 0

     Char. 15: 0 --> 1

     Char. 16: 2 --> 0

     Char. 24: 1 --> 0

     Char. 26: 1 --> 0

     Char. 41: 1 --> 0

     Char. 48: 0 --> 1

     Char. 53: 1 --> 0

     Char. 67: 0 --> 2

     Char. 90: 1 --> 0

     Char. 91: 1 --> 0

*Dobrogeria* :

     Char. 0: 0 --> 1

*Lualabaea* :

     Char. 109: 0 --> 1

*Ticinepomis* :

     Char. 54: 4 --> 3

*Foreyia* :

     Char. 95: 1 --> 0

     Char. 103: 0 --> 1

*Trachymetopon* :

     Char. 69: 0 --> 1

     Char. 81: 1 --> 0

     Char. 95: 1 --> 0

*Atacamaia* :

     Char. 6: 1 --> 0

     Char. 27: 0 --> 1

*Gavinia* :

     No autapomorphies

*Serenichthys* :

     Char. 16: 0 --> 1

     Char. 95: 0 --> 1

*Styloichthys* :

     No autapomorphies

*Heptanema* :

     No autapomorphies

   IMG_2380 :

     No autapomorphies

   YPM_14932 :

     No autapomorphies

   YPM_14933 :

     No autapomorphies

   YPM_29366 :

     No autapomorphies

   YPM_14920 :

     No autapomorphies

   YPM_14921 :

     No autapomorphies

   YPM_14558b :

     No autapomorphies

   YPM_14918a :

     No autapomorphies

   YPM_14941 :

     No autapomorphies

   YPM_14935 :

     No autapomorphies

   YPM_14934 :

     No autapomorphies

   YPM_14929 :

     No autapomorphies

   YPM_14949 :

     Char. 60: 1 --> 0

   YPM_14924 :

     No autapomorphies

   YPM_14939 :

     Char. 48: 0 --> 2

    YPM_14957 :

      No autapomorphies

    YPM_7504 :

      No autapomorphies

    YPM_7516 :

      No autapomorphies

    YPM_14955 :

      Char. 48: 0 --> 2

   Node 66 :

      No synapomorphies

   Node 67 :

      Char. 6: 0 --> 1

      Char. 11: 1 --> 0

      Char. 18: 0 --> 1

      Char. 83: 1 --> 0

      Char. 92: 0 --> 1

      Char. 94: 0 --> 1

   Node 68 :

      Char. 57: 0 --> 1

   Node 69 :

      Char. 32: 1 --> 0

      Char. 87: 0 --> 1

   Node 70 :

      Char. 0: 0 --> 1

      Char. 2: 1 --> 0

      Char. 16: 0 --> 1

      Char. 19: 0 --> 1

      Char. 55: 0 --> 1

      Char. 66: 0 --> 1

   Node 71 :

      Char. 53: 0 --> 1

      Char. 93: 0 --> 1

   Node 72 :

      Char. 100: 0 --> 1

   Node 73 :

      Char. 22: 2 --> 0

      Char. 23: 1 --> 0

      Char. 33: 1 --> 0

      Char. 44: 0 --> 1

   Node 74 :

      Char. 62: 0 --> 1

      Char. 96: 0 --> 1

   Node 75 :

      Char. 100: 1 --> 0

   Node 76 :

      Char. 22: 0 --> 1

      Char. 58: 0 --> 1

      Char. 90: 0 --> 1

   Node 77 :

      Char. 96: 0 --> 1

      Char. 108: 0 --> 1

   Node 78 :

      Char. 0: 1 --> 0

      Char. 20: 0 --> 1

   Node 79 :

      Char. 4: 0 --> 1

      Char. 66: 1 --> 0

      Char. 74: 0 --> 1

   Node 80 :

      Char. 28: 0 --> 1

      Char. 61: 0 --> 1

   Node 81 :

      Char. 22: 0 --> 2

      Char. 33: 0 --> 1

   Node 82 :

      No synapomorphies

   Node 83 :

      Char. 14: 0 --> 1

   Node 84 :

      Char. 7: 1 --> 0

      Char. 35: 0 --> 1

      Char. 95: 0 --> 2

      Char. 96: 1 --> 0

   Node 85 :

      Char. 29: 1 --> 0

      Char. 46: 0 --> 1

      Char. 98: 0 --> 1

   Node 86 :

      Char. 44: 1 --> 0

      Char. 47: 0 --> 1

      Char. 90: 0 --> 1

      Char. 99: 0 --> 1

   Node 87 (Mawsoniidae) :

      Char. 8: 1 --> 0

      Char. 16: 2 --> 1

      Char. 26: 1 --> 2

      Char. 35: 0 --> 1

      Char. 41: 1 --> 0

      Char. 42: 0 --> 1

      Char. 44: 1 --> 0

      Char. 48: 0 --> 2

      Char. 49: 1 --> 0

      Char. 56: 0 --> 1

   Node 88 (Latimeroidea):

      Char. 13: 1 --> 0

      Char. 26: 0 --> 1

      Char. 31: 1 --> 0

      Char. 35: 1 --> 0

   Node 89 :

      No synapomorphies

   Node 90 :

      Char. 34: 0 --> 1

      Char. 51: 1 --> 0

   Node 91 :

      No synapomorphies

   Node 92 :

      Char. 26: 1 --> 0

      Char. 31: 0 --> 1

      Char. 48: 0 --> 1

      Char. 66: 0 --> 1

   Node 93 (Latimeriidae) :

      Char. 5: 0 --> 1

      Char. 13: 0 --> 1

      Char. 29: 1 --> 0

      Char. 56: 0 --> 1

      Char. 59: 0 --> 1

      Char. 78: 0 --> 1

      Char. 91: 1 --> 0

      Char. 102: 0 --> 1

      Char. 103: 1 --> 0

      Char. 109: 0 --> 1

   Node 94 :

      Char. 58: 0 --> 1

      Char. 102: 1 --> 0

   Node 95 :

      Char. 67: 0 --> 1

   Node 96 :

      Char. 0: 0 --> 1

   Node 97 :

      No synapomorphies

   Node 98 :

      Char. 15: 0 --> 1

      Char. 16: 1 --> 0

      Char. 61: 1 --> 0

   Node 99 :

      Char. 29: 1 --> 0

   Node 100 :

      Char. 38: 0 --> 1

      Char. 39: 0 --> 1

      Char. 50: 0 --> 1

      Char. 95: 1 --> 2

   Node 101 :

      Char. 25: 1 --> 0

      Char. 29: 0 --> 1

   Node 102 :

      Char. 6: 1 --> 0

   Node 103 :

      Char. 8: 1 --> 0

      Char. 26: 1 --> 0

      Char. 35: 0 --> 1

      Char. 48: 0 --> 1

      Char. 51: 0 --> 1

   Node 104 :

      Char. 7: 1 --> 0

      Char. 42: 0 --> 1

   Node 105 :

      Char. 30: 1 --> 0

      Char. 61: 0 --> 1

   Node 106 :

      No synapomorphies

   Node 107 :

      No synapomorphies

   Node 108 :

      No synapomorphies

   Node 109 :

      No synapomorphies

   Node 110 :

      No synapomorphies

   Node 111 :

      No synapomorphies

   Node 112 :

      No synapomorphies

   Node 113 :

      Char. 48: 0 --> 2

   Node 114 :

      No synapomorphies

without *D. longicaudatus* YPM VPPU 14555 from Firestone:

CI: 0.355

TL: 348

RI: 0.731

Total MPTS: 18

Tree 18 :

    Actinopterygii :

      No autapomorphies

    †Porolepiformes :

      Char. 11: 1 --> 0

      Char. 66: 0 --> 1

      Char. 79: 0 --> 1

    †*Diplocercides* :

      Char. 79: 0 --> 1

      Char. 82: 0 --> 1

*Rhabdoderma* :

      Char. 45: 0 --> 1

      Char. 76: 1 --> 0

      Char. 77: 1 --> 0

    †*Caridosuctor* :

      No autapomorphies

    †*Hadronector* :

      No autapomorphies

    †*Rebellatrix* :

      No autapomorphies

    †*Polyosteorhynchus* :

      Char. 20: 0 --> 1

      Char. 96: 0 --> 1

    †*Allenypterus* :

      Char. 58: 0 --> 1

    †*Lochmocercus* :

      Char. 20: 0 --> 1

    †*Coelacanthus* :

      Char. 3: 0 --> 1

      Char. 9: 1 --> 0

      Char. 25: 0 --> 1

      Char. 36: 0 --> 1

      Char. 41: 0 --> 1

      Char. 46: 0 --> 1

    †*Spermatodus* :

      Char. 14: 0 --> 1

      Char. 25: 0 --> 1

      Char. 69: 1 --> 0

    †*Whiteia* :

      Char. 4: 1 --> 0

      Char. 45: 0 --> 1

      Char. 83: 0 --> 1

    †*Laugia* :

      Char. 6: 1 --> 0

      Char. 25: 0 --> 1

      Char. 30: 1 --> 0

      Char. 55: 1 --> 0

    †*Sassenia* :

     Char. 68: 1 --> 0

   †*Chinlea* :

     Char. 66: 0 --> 1

     Char. 97: 1 --> 0

     Char. 105: 0 --> 1

   †*Holophagus* :

     Char. 0: 0 --> 1

     Char. 98: 0 --> 1

   †*Undina* :

     Char. 4: 1 --> 0

     Char. 62: 1 --> 0

   †*Coccoderma* :

     Char. 22: 0 --> 2

     Char. 26: 0 --> 1

     Char. 36: 0 --> 1

     Char. 37: 0 --> 1

     Char. 48: 1 --> 0

     Char. 88: 0 --> 1

     Char. 96: 1 --> 0

     Char. 105: 0 --> 1

   †*Libys* :

     No autapomorphies

  †*Mawsonia* :

     Char. 16: 0 --> 3

     Char. 34: 1 --> 0

     Char. 70: 0 --> 1

   †*Macropoma* :

     Char. 1: 0 --> 2

     Char. 66: 1 --> 0

     Char. 88: 0 --> 1

     Char. 103: 0 --> 1

   †*Latimeria* :

     Char. 3: 0 --> 1

     Char. 45: 0 --> 1

     Char. 49: 0 --> 1

     Char. 106: 1 --> 0

   †*Miguashaia* :

     Char. 65: 0 --> 1

   †*Axelrodichthys* :

     Char. 19: 1 --> 0

     Char. 37: 0 --> 1

   †*Holopterygius* :

     Char. 92: 1 --> 0

     Char. 98: 0 --> 1

   †*Garnbergia* :

     Char. 97: 1 --> 0

   †*Parnaibaia* :

     Char. 2: 0 --> 1

     Char. 3: 0 --> 1

     Char. 14: 0 --> 1

     Char. 25: 1 --> 0

     Char. 77: 0 --> 1

   †*Swenzia* :

     Char. 1: 0 --> 1

   †*Guizhoucoelacanthus* :

     Char. 16: 1 --> 0

     Char. 24: 1 --> 0

     Char. 42: 0 --> 1

     Char. 51: 1 --> 0

     Char. 94: 1 --> 0

     Char. 105: 0 --> 1

   †*Piveteauia* :

     Char. 12: 0 --> 1

     Char. 21: 0 --> 1

     Char. 45: 0 --> 1

     Char. 52: 0 --> 1

     Char. 78: 0 --> 1

   †*Axelia* :

     Char. 67: 0 --> 1

   †*Wimania* :

     No autapomorphies

   †*Megalocoelacanthus* :

     Char. 4: 1 --> 0

     Char. 55: 1 --> 0

   †*Luopingcoelacanthus* :

     Char. 34: 1 --> 0

   †*Yunnancoelacanthus* :

     Char. 15: 0 --> 1

     Char. 16: 1 --> 0

     Char. 35: 1 --> 0

     Char. 53: 1 --> 0

     Char. 56: 1 --> 0

     Char. 67: 0 --> 2

   †*Dobrogeria* :

     Char. 0: 0 --> 1

   †*Lualabaea* :

     Char. 109: 0 --> 1

   †*Ticinepomis* :

     Char. 54: 4 --> 3

   †*Foreyia* :

     Char. 95: 1 --> 0

     Char. 103: 0 --> 1

   †*Trachymetopon* :

     Char. 64: 1 --> 0

     Char. 69: 0 --> 1

     Char. 81: 1 --> 0

     Char. 95: 1 --> 0

   †*Atacamaia* :

     Char. 6: 1 --> 0

     Char. 27: 0 --> 1

     Char. 41: 0 --> 1

  † *Gavinia* :

     No autapomorphies

   †*Serenichthys* :

     Char. 16: 0 --> 1

     Char. 95: 0 --> 1

   †*Styloichthys* :

     No autapomorphies

   †*Heptanema* :

     Char. 48: 1 --> 0

   IMG_2380 :

     No autapomorphies

   YPM_14932 :

     No autapomorphies

   YPM_14933 :

     No autapomorphies

   YPM_29366 :

     No autapomorphies

   YPM_14920 :

     No autapomorphies

   YPM_14921 :

     No autapomorphies

   YPM_14558b :

     No autapomorphies

   YPM_14918a :

     No autapomorphies

   YPM_14941 :

     No autapomorphies

   YPM_14935 :

     No autapomorphies

   YPM_14934 :

     No autapomorphies

   YPM_14929 :

     No autapomorphies

   YPM_14949 :

     Char. 48: 0 --> 2

     Char. 60: 1 --> 0

   YPM_14924 :

     Char. 48: 0 --> 2

   YPM_14939 :

     Char. 48: 0 --> 2

   YPM_14957 :

     No autapomorphies

   YPM_7504 :

      No autapomorphies

    YPM_7516 :

      Char. 48: 0 --> 2

   Node 65 :

      No synapomorphies

   Node 66 :

      Char. 6: 0 --> 1

      Char. 11: 1 --> 0

      Char. 18: 0 --> 1

      Char. 83: 1 --> 0

      Char. 92: 0 --> 1

      Char. 94: 0 --> 1

   Node 67 :

      Char. 57: 0 --> 1

   Node 68 :

      Char. 32: 1 --> 0

      Char. 87: 0 --> 1

   Node 69 :

      Char. 0: 0 --> 1

      Char. 2: 1 --> 0

      Char. 16: 0 --> 1

      Char. 19: 0 --> 1

      Char. 55: 0 --> 1

      Char. 66: 0 --> 1

   Node 70 :

      Char. 53: 0 --> 1

      Char. 93: 0 --> 1

   Node 71 :

      Char. 100: 0 --> 1

   Node 72 :

      Char. 22: 2 --> 0

      Char. 23: 1 --> 0

      Char. 33: 1 --> 0

      Char. 44: 0 --> 1

   Node 73 :

      Char. 62: 0 --> 1

      Char. 96: 0 --> 1

   Node 74 :

      Char. 100: 1 --> 0

   Node 75 :

      Char. 22: 0 --> 1

      Char. 58: 0 --> 1

      Char. 90: 0 --> 1

   Node 76 :

      Char. 96: 0 --> 1

      Char. 108: 0 --> 1

   Node 77 :

      Char. 0: 1 --> 0

      Char. 20: 0 --> 1

   Node 78 :

      Char. 4: 0 --> 1

      Char. 66: 1 --> 0

      Char. 74: 0 --> 1

   Node 79 :

      Char. 28: 0 --> 1

      Char. 61: 0 --> 1

   Node 80 :

      Char. 22: 0 --> 2

      Char. 33: 0 --> 1

   Node 81 :

      Char. 56: 0 --> 1

   Node 82 :

      Char. 14: 0 --> 1

   Node 83 :

      Char. 7: 1 --> 0

      Char. 35: 0 --> 1

      Char. 95: 0 --> 2

      Char. 96: 1 --> 0

   Node 84 :

      Char. 29: 1 --> 0

      Char. 46: 0 --> 1

      Char. 98: 0 --> 1

   Node 85 :

      Char. 44: 1 --> 0

      Char. 47: 0 --> 1

      Char. 90: 0 --> 1

      Char. 99: 0 --> 1

   Node 86 :

      Char. 9: 1 --> 0

      Char. 26: 0 --> 2

      Char. 48: 1 --> 2

      Char. 91: 0 --> 1

   Node 87 :

      Char. 14: 1 --> 0

      Char. 24: 1 --> 0

   Node 88 (Mawsoniidae) :

      Char. 42: 0 --> 1

      Char. 44: 1 --> 0

      Char. 103: 0 --> 1

   Node 89 (Latimeroidea):

      Char. 31: 1 --> 0

      Char. 95: 2 --> 1

   Node 90 :

      Char. 12: 0 --> 1

      Char. 25: 0 --> 1

      Char. 34: 0 --> 1

      Char. 51: 1 --> 0

   Node 91 :

      Char. 8: 0 --> 1

   Node 92 :

      Char. 31: 0 --> 1

      Char. 66: 0 --> 1

   Node 93 (Latimeriidae crownward of †*Diplurus*) :

      Char. 5: 0 --> 1

      Char. 29: 1 --> 0

      Char. 59: 0 --> 1

      Char. 78: 0 --> 1

      Char. 102: 0 --> 1

      Char. 109: 0 --> 1

   Node 94 (Latimeriidae crownward of †*Garnbergia*):

      Char. 35: 1 --> 0

   Node 95 (Latimeriidae) :

      Char. 9: 1 --> 0

   Node 96 :

      Char. 58: 0 --> 1

      Char. 102: 1 --> 0

   Node 97 :

      Char. 49: 0 --> 2

      Char. 67: 0 --> 1

   Node 98 :

      Char. 0: 0 --> 1

      Char. 105: 0 --> 1

   Node 99 :

      Char. 15: 0 --> 1

      Char. 16: 1 --> 0

      Char. 61: 1 --> 0

   Node 100 :

      Char. 29: 1 --> 0

      Char. 55: 1 --> 0

      Char. 64: 0 --> 1

   Node 101 :

      Char. 38: 0 --> 1

      Char. 39: 0 --> 1

      Char. 50: 0 --> 1

      Char. 95: 1 --> 2

   Node 102 :

      Char. 25: 1 --> 0

      Char. 29: 0 --> 1

   Node 103 :

      Char. 6: 1 --> 0

   Node 104 :

      Char. 51: 0 --> 1

   Node 105 :

      Char. 7: 1 --> 0

     Char. 41: 0 --> 1

      Char. 42: 0 --> 1

      Char. 106: 1 --> 0

   Node 106 :

      Char. 30: 1 --> 0

      Char. 61: 0 --> 1

   Node 107 (†*Diplurus*) :

      Char. 8: 0 --> 1

      Char. 13: 1 --> 0

      Char. 41: 0 --> 1

      Char. 45: 0 --> 1

      Char. 49: 0 --> 1

      Char. 55: 1 --> 0

      Char. 56: 1 --> 0

      Char. 91: 0 --> 1

      Char. 103: 0 --> 1

      Char. 104: 1 --> 0

      Char. 106: 1 --> 0

1. **Supplementary Figure Captions.**

**Figure S1. Parsimony trees.** Strict consensus (a, c) and resampled trees (b, d) from (a, b) the analysis including and (c, d) the analyses excluding †*Diplurus longicaudatus* YPM VPPU 14555.
