# Supplementary figures and images for: Species delimitation and coexistence in an ancient, depauperate vertebrate clade
Source: BMC Ecol Evol. 2022 Jul 12;22:90. doi: 10.1186/s12862-022-02043-4 (PMC9277872; doi:10.1186/s12862-022-02043-4)

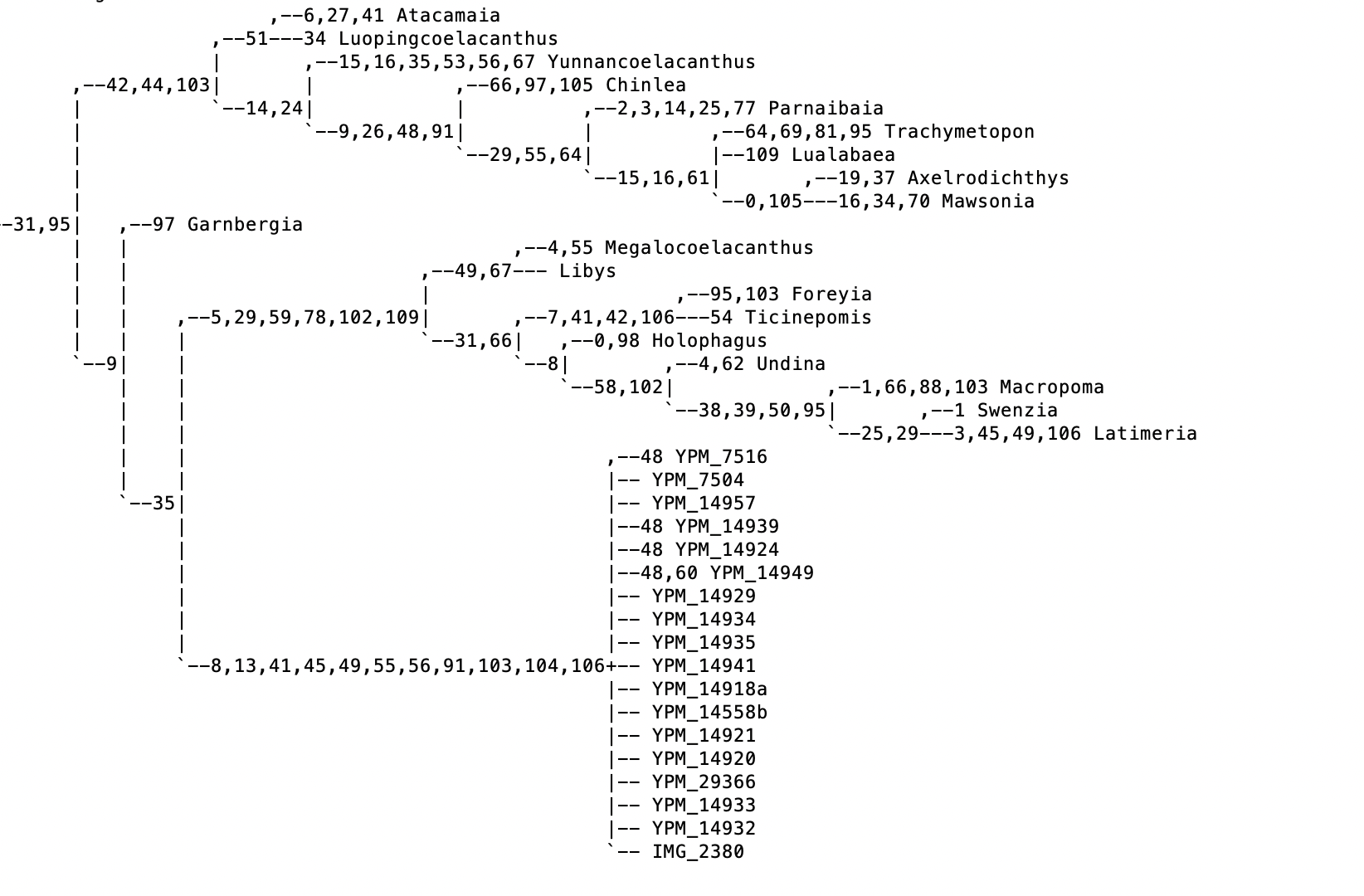

Supplement: Supplementary file 3 — Additional file 3. Phylogenetic data. [file 12862_2022_2043_MOESM3_ESM.zip › Phylogeny/parsimony/Screen Shot 2021-12-26 at 11.36.16 AM.png]

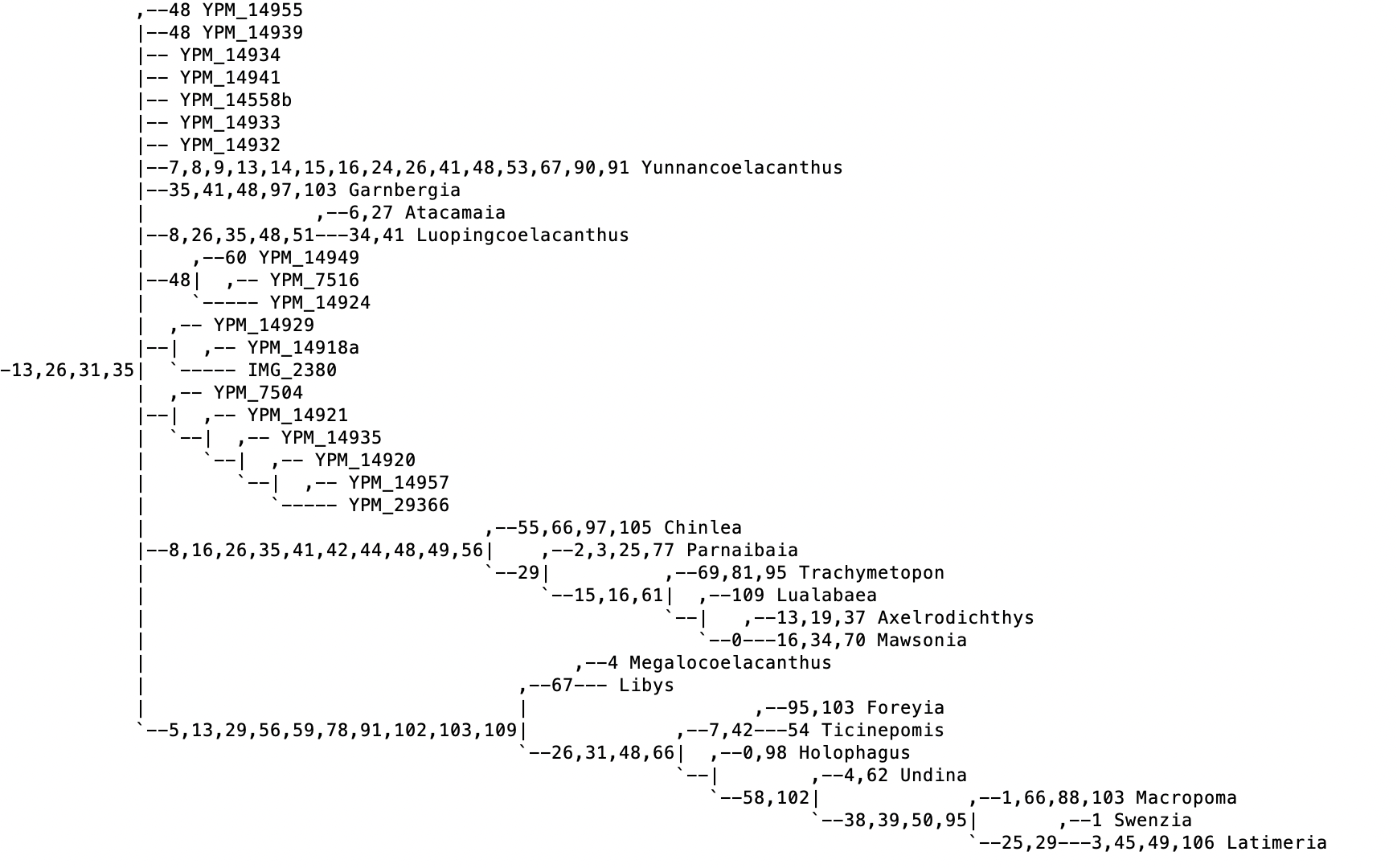

Supplement: Supplementary file 3 — Additional file 3. Phylogenetic data. [file 12862_2022_2043_MOESM3_ESM.zip › Phylogeny/parsimony/Screen Shot 2021-12-25 at 10.28.38 PM.png]
